# Efficacy of Incentive Spirometer in Increasing Maximum Inspiratory Volume in an Out-Patient Setting

**DOI:** 10.7759/cureus.18483

**Published:** 2021-10-04

**Authors:** Harjyot Toor, Samir Kashyap, Anson Yau, Mishel Simoni, Saman Farr, Paras Savla, Robert Kounang, Dan E Miulli

**Affiliations:** 1 Neurosurgery, Riverside University Health System Medical Center, Moreno Valley, USA; 2 Pain Management, Arrowhead Regional Medical Center, Colton, USA; 3 Neurosurgery, Riverside University Health System Medical Center, Riverside, USA; 4 Neurosurgery, Arrowhead Regional Medical Center, Colton, USA

**Keywords:** incentive spirometer, pulmonary function, maximum inspiratory volume, chronic pain, out-patient clinic

## Abstract

Background

Incentive spirometry (IS) is the mainstay of care in postoperative patients that has been heavily studied in the inpatient setting. Studies have shown that the utilization of IS improves lung volumes and reduces the rate of pneumonia in post-surgical patients. However, the literature is ambiguous on its benefit as many studies also demonstrate no significant benefit, especially in comparison to early ambulation. Our study sought to determine whether a consistent IS regimen can improve lung function in an outpatient setting.

Methods

This prospective cohort study included patients in a physical medicine and rehabilitation clinic setting during the COVID pandemic. Patients with severe respiratory disease, baseline cough, those unable to perform deep breathing, fever greater than 100.4 F due to non-pulmonary on initial evaluation, or inability to fill out the forms and complete the study were excluded. Each participant was given the IS along with hands-on instruction on how to use the device and accurately record measurements. Patients were asked to lie down and inhale and exhale through the tube ten times. They were asked to mark the highest volume during their 10 breaths. Patients were instructed to complete this exercise three times a day for 30 days. Patients were also asked to perform light exercises or walking for 20 minutes per day three times a week and postural drainage. Patients were instructed to call their primary care physician if a 20% or more decrease from their baseline was noted or if they experienced any new coughs, fever, or shortness of breath during the 30 days of exercise.

Results

A total of 48 patients enrolled in the study with a (median) age of 58.0 years (SD 10.2 years), 21 females and 27 males. Baseline maximal inspiration for study participants was 1885.4 mL prior to exercise, with a subsequent increase in lung capacity observed for all participants enrolled in the study. At the end of the study period, week four, the average maximal inspiratory volume was 2235.4 mL. Paired t-test showed a significant difference between baseline (1885.4) and maximum (2235.4) volumes (t=-4.59, p<0.0001). Analysis of variance (ANOVA) showed no significant difference among Week 1-4 averages (F=1.08, p=0.36). None of the participants reported any symptoms (fever, coughing, shortness of breath) or COVID-19 infection during the 30-days period. None of the participants reported contacting primary care physicians.

Conclusion

When prescribed daily breathing exercises with an incentive spirometer, study participants experienced a 16% increase in maximal inspiratory volume over a span of 30 days and did not need to contact their primary care physician during the study period.

## Introduction

Patients with chronic pain conditions may have impaired deep breathing. These patients are at risk for respiratory dysfunction due to various causes such as pneumonia, atelectasis, bronchiectasis, sleep apnea, chronic obstructive lung disease, and restrictive lung disease. In addition, patients with poor nutritional status, drooling, aspiration, gastroesophageal reflux, impairment of airway clearance due to muscular weakness or incoordination, and poor pulmonary reserve are at higher risk of respiratory insufficiency, morbidity, and mortality.

Incentive spirometer (IS) is the mainstay of care in postoperative and hospitalized patients that has been heavily studied in the inpatient setting [1-3]. It encourages the patient to perform slow and deep inspiration through visual feedback, allowing for the stretching and opening of collapsed airways. Incentive spirometer exercise is useful as it is inexpensive, simple to use, with no known side effects, and does not require supervision once the patient is trained in its use. Furthermore, achievement of the visual target encourages the patient to try their best and thus promotes patient compliance. 

Studies have shown that utilization of IS improves lung volumes and reduces rates of pneumonia in post-surgical patients [2,4,5]. It has also demonstrated utility in patients with a variety of conditions and age groups. Elseify et al. found significant improvements in forced vital capacity and forced expiratory volume in children with cerebral palsy [6]. Another study found significant improvements in pulmonary function in those with spinal cord injury [7]. Our study sought to determine whether a consistent IS regimen resulted in improved lung function in an outpatient setting and whether IS can be used by the patient as a signal to notify the primary care physician when goals are not met or related symptoms begin.

## Materials and methods

Patients screened for inclusion into our study were patients from a physical medicine and rehabilitation clinic. All patients in outpatient clinics were given the opportunity to enroll in the study. Only people presenting with severe respiratory disease, baseline cough, those unable to perform deep breathing, or fever greater than 100.4 F due to non-pulmonary etiology on initial evaluation were excluded. The intake sheet included gender, height, weight, smoking, chronic obstructive pulmonary disease (COPD), asthma or other medical history, medications used, and baseline temperature.

Patients were given the incentive spirometer free of charge, along with hands-on instruction on how to use the device and accurately record measurements. Patients were asked to lie down and were instructed to inhale through their mouth slowly over 10 seconds, exhale completely and repeat this 10 times consecutively. There was no recording available for exhalation. The patients were instructed to mark the highest inspired volume during their 10 breaths by sliding the plastic marker on the device. Patients were instructed to complete this exercise three times a day for 30 days. During the first encounter, patients’ baseline volumes were also recorded by research assistants and marked on the incentive spirometer. If a 20% or more decrease from their baseline was noted, patients were asked to call their primary care physician. Follow-up questions included whether they experienced any new coughs, fever, or shortness of breath during the 30 days of exercise.

Patients were also asked and committed to general cardiopulmonary fitness and endurance training through walking or light jogging to achieve 70% targeted heart rate for 20 minutes three times a week. For those individuals who were not able to sustain or tolerate high activity, we discussed substituting three minutes of walking or light jogging to achieve 70% targeted heart rate alternating with equal periods of rest, for a cumulative of 30 minutes. Participants were also taught abdominal muscle strengthening exercises to improve fitness and improve the ability to clear secretions.

Furthermore, the participants were instructed to perform postural drainage exercises to enhance the clearance of phlegm by using gravity and position changes. They were instructed to apply percussion to the back, chest, or sides. Patients were requested to perform postural drainage in the morning before eating, on the bed or floor, with the chest lower than the hips to allow mucus to drain. They were also instructed to use pillows, foam wedges, and other devices for comfort and to elevate the hips above the chest. Patients were instructed to first perform the exercise on the back, then each side, then on the stomach, holding each position for a minimum of five minutes. During each position, they were instructed to breathe deeply and slowly in through the nose and out through the mouth. Patients were informed that postural drainage could be enhanced by another person gently cupping hands and clapping quickly on the side of the chest, side, or back that is facing up during the five minutes. All study participants were compliant with instruction. 

Equipment

250 incentive spirometers were donated through Inland Empire Health Plan (IEHP) and Arrowhead Regional Medical Center (ARMC) Foundation and distributed through the Physical Medicine & Rehabilitation (PM&R) clinic and Family Medicine clinic at ARMC. Overall, 48 patients, or 19%, participated in the study for 30 days, and 48 datasheets were obtained. 

## Results

48 study participants were enrolled in the study with 100% compliance to the 30 day trial period (27 male, 21 female). Study participants performed light exercises daily and completed postural drainage. No study participant reported coughing more than five times during IS use and a need to contact their primary care provider for respiratory issues during the study and one month after the study completion. The average age of the patients was 58.3 years (SD 9.8 years). The median maximum inspiratory volume achieved can be seen in Table 1.

**Table 1:** Median maximum inspiratory volume achieved Volumes in mL

	Baseline	Highest	Week 1	Week 2	Week 3	Week 4
Median	1885.4	2235.4	1925.5	2009.8	2093.3	2184.5
Standard deviation	619.5	549.9	541.9	582.6	517.9	451.8

Paired t-test showed a significant difference between baseline (1885.4) and highest (2235.4) volumes (t=-4.59, p<0.0001). Analysis of variance (ANOVA) showed no significant difference among Week 1-4 averages (F=1.08, p=0.36). None of the participants reported any new symptoms (fever, coughing, shortness of breath) or COVID-19 infection during the 30-days period. None of the participants reported the need to contact their primary care provider during the trial period and 30 days afterward. Patients were encouraged to continue IS during the COVID pandemic.

## Discussion

An incentive spirometer (IS) is a device designed to achieve and sustain maximal inspiration with active recruitment of the diaphragm and other inspiratory muscles. It encourages deep inspiratory and expiratory breathing by having the patient take long, deep breaths with subsequent pauses. This maneuver emphasizes lung inflation, increasing tidal volume, and maintaining patency of the smaller airway [8]. Like other prophylactic measures, such as smoking cessation, administration of antibiotics, bronchodilators, and chest physical therapy, incentive spirometer has been shown to decrease the rate of postoperative lung atelectasis and decrease pulmonary complications after cardiac, lung, or abdominal surgery [8]. It also reduced pulmonary complications, and improved pulmonary function test (PFT) results in patients with rib fractures [8-10]. 

Maximal Inspiratory volume reflects diaphragm strength and ventilation ability. Maximum expiratory pressure is indicative of abdominal and chest wall muscle strength and the ability to cough and clear secretions. Peak cough flow is reported as a simple measurement that indicates the amount of pressure a patient can generate during a voluntary cough. Reports indicate that maximal inspiratory mouth volume and maximal expiratory pressure exclude significant weakness of the respiratory muscles [11]. Patients with a low maximal expiratory pressure have difficulty generating sufficient cough to clear respiratory secretion. When the amount of air forced from the lungs in one second or forced expiratory volume (FEV1) is greater than two liters or 50% of predicted, major complications are rare.

Consequently, this expands the current preventative use of the incentive spirometer to now include the outpatient, ambulatory clinic setting targeting at-risk patient populations for atelectasis, pneumonia, and respiratory infection. Patients are considered high risk if maximal inspiratory mouth pressure and maximal expiratory pressure decrease more than 20% below the patient’s baseline or when the FEV1 becomes less than two liters or 50% of predicted. Patients are predisposed to these conditions with older age, difficulty swallowing, decreased ambulation, swallow breathing, recent general anesthesia or opioid use, and may benefit from incentive spirometry training and use, general cardiopulmonary fitness, and chest postural drainage.

The incentive spirometer is easy to use and provides the patient with visual feedback on airflow and volume. Its use results in a prolonged phase of effective inspiration, more controlled flow, and greater enthusiasm to practice [12]. To prevent decompensation, individuals should practice routine pulmonary exercise training with the goal of achieving increased maximal inspiratory mouth pressure for sex, height, and weight, an increased lung volume expiration, and increased cough capacity. Training should consist of diaphragm, abdominal, and chest wall strengthening through deep inspiration and expiration, as well as cardiopulmonary fitness and endurance training. 

Using an incentive spirometer teaches a patient to take slow, deep breaths and can be helpful to maximize lung capacity after surgery or in the setting of progressive pulmonary disease. Numerous studies have repeatedly demonstrated that simple instruction of performing 10 deep inspirations per hour while awake can dramatically improve lung function and decrease pulmonary complications such as pneumonia and atelectasis. However, currently, there is limited utility for this excellent prophylactic tool. This study demonstrates that IS can be incorporated into a health regimen for at-risk populations. 

The results in our study are encouraging; however, there are limitations. Designed as a pilot investigation, our study had a small study size of 48 patients. A subsequent, larger, prospective study will need to be conducted with higher patient recruitment to assess the reliability and reproducibility of the results. 

Furthermore, due to Covid 19 social distancing constraints implemented at the time of the study, patient follow-up was limited to telephone and surveys. After initial instruction was provided to the study participants, and follow-up was requested at one, two, three, and four weeks no additional in-person instruction or assessment was performed to ensure participants were correctly utilizing the incentive spirometer. Patients may have an incorrect understanding and device utilization, although they believed they were correct as documented through weekly phone contact. Virtual visits were not able to be set up system-wide, which would have permitted visual confirmation of understanding. Eltorai et al. recommend an intermittent reassessment of patient performance when using the IS after initial instruction to ensure correct utilization of the device. However, the amount of time that providers spend on IS-related activities has not been reported, nor have optimal use procedures been established [13]. 

## Conclusions

The present study demonstrated that when prescribed daily breathing exercises with an incentive spirometer, instructions for general cardiopulmonary fitness, and chest postural drainage during the COVID pandemic, ambulatory clinic patients experienced a significant 16% increase in maximal inspiratory volume over a 30-day period without developing a decreased inspiration volume, cough, fever to greater than 100.4 F, or a need to call their primary care physician for respiratory complaints.
